# Identifying the Main Mosquito Species in China Based on DNA Barcoding

**DOI:** 10.1371/journal.pone.0047051

**Published:** 2012-10-10

**Authors:** Gang Wang, Chunxiao Li, Xiaoxia Guo, Dan Xing, Yande Dong, Zhongming Wang, Yingmei Zhang, Meide Liu, Zhong Zheng, Hengduan Zhang, Xiaojuan Zhu, Zhiming Wu, Tongyan Zhao

**Affiliations:** 1 Institute of Microbiology and Epidemiology, Academy of Military Medical Science, Beijing, China; 2 Center for Disease Control and Prevention of Military Area Command, Xinjiang, China; Instituto de Higiene e Medicina Tropical, Portugal

## Abstract

Mosquitoes are insects of the Diptera, Nematocera, and Culicidae families, some species of which are important disease vectors. Identifying mosquito species based on morphological characteristics is difficult, particularly the identification of specimens collected in the field as part of disease surveillance programs. Because of this difficulty, we constructed DNA barcodes of the cytochrome c oxidase subunit 1, the COI gene, for the more common mosquito species in China, including the major disease vectors. A total of 404 mosquito specimens were collected and assigned to 15 genera and 122 species and subspecies on the basis of morphological characteristics. Individuals of the same species grouped closely together in a Neighborhood-Joining tree based on COI sequence similarity, regardless of collection site. COI gene sequence divergence was approximately 30 times higher for species in the same genus than for members of the same species. Divergence in over 98% of congeneric species ranged from 2.3% to 21.8%, whereas divergence in conspecific individuals ranged from 0% to 1.67%. Cryptic species may be common and a few pseudogenes were detected.

## Introduction

Approximately 41 genera and 3500 species and subspecies of mosquito exist worldwide. Although mosquitoes have been studied more extensively than most other insect groups because of their role as vectors of disease, our taxonomic knowledge of these insects is far from complete. Numerous Chinese taxonomists have worked on mosquito classification since 1932, particularly since Edwards provided the modern mosquito classification system [1]. Feng Lan-Zhou reported 100 Chinese mosquito species in 1938 [2]. This number has since then increased to approximately 390 described species and new species are still being identified, particularly within the genera *Armigeres*, *Heizmannia*, *Topomyia* and *Uranotaenia*.

Some species are vectors of medically important pathogens, such as malaria, Dengue fever and Japanese B encephalitis. Species identification therefore constitutes the first step in the surveillance and control of mosquito-borne diseases. The identification of mosquito species is mainly done on the basis of morphological characteristics. This can be problematic because diagnostic morphological features are often damaged during collection or storage, or are not present in all developmental stages. Moreover, the morphological characteristics used to identify intact adult specimens often vary so little between species that usually only experienced mosquito taxonomists are able to distinguish mosquito species reliably [3].

DNA analysis provides a more accurate way of identifying species and the use of molecular data, in combination to morphological methods, has resolved some long-standing taxonomic questions [4], [5]. The increase in the number of available molecular markers has facilitated the accurate identification of mosquito species, particularly within groups of sibling species. For instance, *Anopheles anthropophagus* and *Anopheles sinensis* can be identified more simply, rapidly, and accurately using the ITS2 sequence than on the basis of morphology [6], [7].

After Tautz proposed using DNA sequences as the main basis of biological classification in 2002 [8], [9] Paul Hebert suggested that sequencing the COI gene could allow DNA barcoding that would facilitate such classification [10]–[12]. Many studies have since then demonstrated that the COI gene is a valid molecular tool for identifying mosquito species [13], [14] and revealing cryptic species [15]–[18].

Although several studies on the distribution of Chinese mosquito species have been conducted using classical morphology identifying sibling and cryptic species remains problematic. Here we provide an updated classification of nearly one-third of China’s mosquito species based on a combination of molecular and morphological methods.

## Results

### Specimen Collection

**Figure 1:**
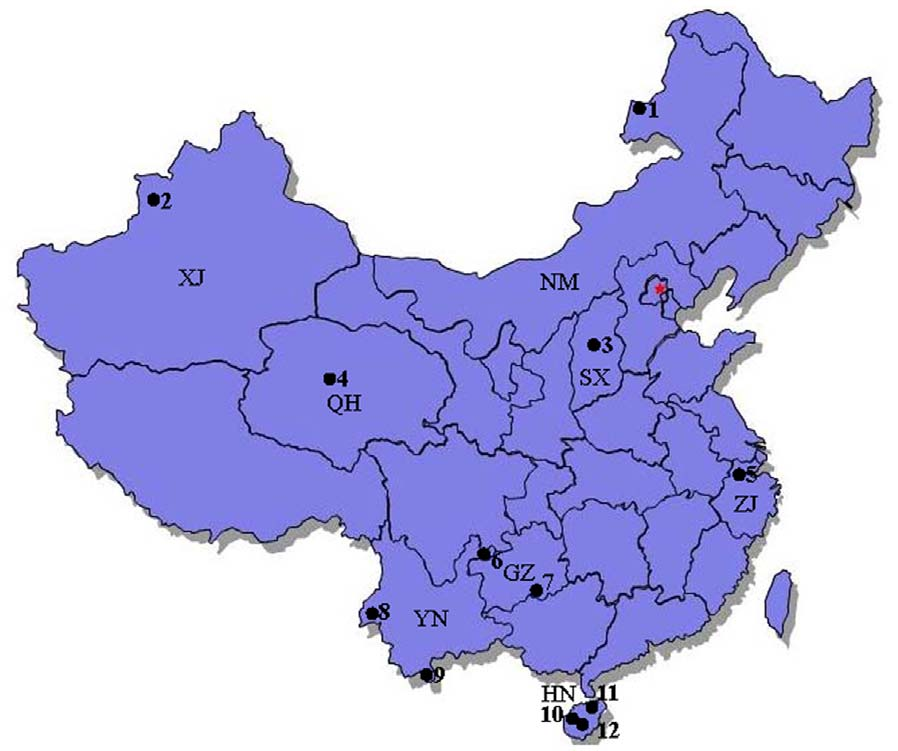
Map of the study area showing the sampling sites of mosquitoes collected in this study. Site 1: Manzhouli City, Neimeng ProvinceXinjiang; Site 2: Yili, Kazakh Autonomous Prefecture, Xinjiang Province; Site 3: Taiyuan City, Shanxi Province; Site 4: Golmud River, QinghaiQinghai Province; Site 5: Tianmu Mountain, Zhejiang Province; Site 6: Zhenxiong County, Yunnan Province; Site 7: Maolan Natural Reserve, Guizhou Province; Site 8: Ruili City, Yunnan Province; Site 9: Mengla County, Yunnan Province; Site 10: Changjiang County, Hainan Province; Site 11: Limushan Nature Reserve, Hainan Province; Site 12: Mangrove Nature Reserve, Hainan Province.

A total of 122 mosquito species belonging to 15 genera and three subfamilies were collected from sampling sites in eight Chinese provinces (Figure 1, Table 1). We identified mosquitoes on the basis of diagnostic morphological characteristics of their adult and larval stages and cercopoda [19], and by using molecular methods to distinguish sibling species [6], [7].

**Table 1:** List of mosquito species, collection sites and GenBank accession numbers.

Mosquito species	Collection site	GenBank accession number
*An. lindesayi*	Site 6, Yunnan	JQ728147; JQ728148;JQ728149
	Site 7, Guizhou	JQ728370
	Site 5, Zhejiang	JQ728076
*An. gigas baileyi*	Site 6, Yunnan	JQ728161;JQ728162;JQ728163
*An. barbirostris*	Site 8, Site 9, Yunnan	JQ728025;JQ728220
	Site 10–12, Hainan	JQ728403;JQ728404;JQ728405
*An. barbumbrosus*	Site 9, Yunnan	JQ728212
*An. jamesii*	Site 9, Yunnan	JQ728209
*An. messeae*	Site 1, Neimeng	JQ728113; JQ728114; JQ728115; JQ728116; JQ728077
	Site 2, Xinjiang	JQ728279; JQ728280
*An. sinensis*	Site 6, Site 8, Site 9, Yunnan	JQ728141;JQ728388;JQ728389; JQ728390; JQ728391;JQ728343; JQ728233
	Site 10–12, Hainan	JQ728409; JQ728410;JQ728411
	Lab	JQ728020
*An. yatsushiroensis*	Site 3, Shanxi	JQ728372; JQ728373
*An. hyrcanus*	Site 2, Xinjiang	JQ728293; JQ728294;JQ728295
*An. claviger*	Site 2, Xinjiang	JQ728274
*An. kweiyangensis*	Site 6, Yunnan	JQ728386
	Site 5, Zhejiang	JQ728378
*An.sawadwongpormi*	Site 12, Hainan	JQ728407; JQ728408
*An. peditaeniatus*	Site 8, Site 9, Yunnan	JQ728088; JQ728089;JQ728090; JQ728342; JQ728230; JQ728231
*An. maculatus*	Site 9, Yunnan	JQ728164
*An. xui*	Site 9, Yunnan	JQ728232; JQ728203
*An. tessellatus*	Site 8, Site 9, Yunnan	JQ728102; JQ728103
	Site 10–12, Hainan	JQ728050; JQ728051; JQ728052; JQ728053;JQ728054
*An. kochi*	Site 8, Site 9, Yunnan	JQ728307; JQ728242;JQ728243; JQ728290; JQ728291; JQ728292
*An. aitkenii*	Site 9, Yunnan	JQ728268;JQ728269; JQ728270
*An. pseudowillmori*	Site 9, Yunnan	JQ728241
*An. vagus*	Site 8, Site 9, Yunnan	JQ728070; JQ728042
	Site 10–12, Hainan	JQ728305; JQ728045; JQ728044; JQ728043
*An. minimus*	Site 9, Yunnan	JQ728026; JQ728027; JQ728028; JQ728029
	Site 10, Hainan	JQ728406; JQ728030
*An. aconitus*	Site 9, Yunnan	JQ728412; JQ728413; JQ728414; JQ728415; JQ728416
	Site 10, Hainan	JQ728306; JQ728417; JQ728418; JQ728419
*An. jeyporiensis*	Site 9, Yunnan	JQ728235; JQ728236; JQ728218
*An. dirus*	Site 12, Hainan	JQ728302; JQ728303
*An. splendidus*	Site 8, Yunnan	JQ728261
*Cx. halifaxia*	Site 9, Yunnan	JQ728180; JQ728387; JQ728333
	Site 10, Hainan	JQ728073; JQ728074; JQ728075
*Cx. brevipalpis*	Site 8, Site 9, Yunnan	JQ728158; JQ728159; JQ728160; JQ728336
	Site 7, Guizhou	JQ728358; JQ728359
*Cx. foliatus*	Site 9, Yunnan	JQ728234
*Cx. minor*	Site 9, Yunnan	JQ728188; JQ728189
	Site 12, Hainan	JQ728374
*Cx. infantulus*	Site 8, Yunnan	JQ728267
*Cx. malayi*	Site 5, Zhejiang	JQ728092
*Cx. richei*	Site 5, Zhejiang	JQ728091; JQ728265
*Cx. peytoni*	Site 9,Yunnan	JQ728379; JQ728380
*Cx. spiculosus*	Site 8, Site 9, Yunnan	JQ728022; JQ728023; JQ728024
*Cx. bicornutus*	Site 9, Yunnan	JQ728205
*Cx. fuscocephala*	Site 8, Site 9, Yunnan	JQ728383; JQ728338; JQ728339; JQ728237; JQ728354
*Cx. hayashii*	Site 9, Yunnan	JQ728264; JQ728266
*Cx. fuscanus*	Site 9, Yunnan	JQ728037
*Cx. rubithoracis*	Site 9, Yunnan	JQ728155
*Cx. infula*	Site 9, Yunnan	JQ728199
*Cx. nigropunctatus*	Site 8, Site 9, Yunnan	JQ728087;JQ728347;JQ728348; JQ728206; JQ728207; JQ728208 JQ728071; JQ728072
	Site 10, Hainan	JQ728298
*Cx. pipiens*	Site 2, Xinjiang	JQ728284; JQ728285; JQ728286
	Lab	JQ728036; JQ728035
*Cx.pipiens quinquefasciatus*	Site 6, Site 8–9, Yunnan	JQ728381;JQ728382;JQ728327
	Lab	JQ728021
*Cx. pipiens pallens*	Lab	JQ728040
*Cx. pallidothorax*	Site 10, Hainan	JQ728057; JQ728058
*Cx. whitmorei*	Site 9, Yunnan	JQ728304
*Cx.bitaeniorhynchus*	Site 8–9, Yunnan	JQ728034; JQ728349; JQ728200
*Cx. sitiens*	Site 10, Hainan	JQ728396; JQ728397; JQ728398; JQ728399; JQ728400; JQ728401; JQ728402
*Cx. mimulus*	Site 9, Yunnan	JQ728244; JQ728245; JQ728246; JQ728247
	Site 5, Zhejiang	JQ728082; JQ728083; JQ728084; JQ728085; JQ728086
*Cx. mimeticus*	Site 9, Yunnan	JQ728150; JQ728151; JQ728152
	Site 5, Zhejiang	JQ728078
*Cx. murrelli*	Site 5, Zhejiang	JQ728079; JQ728080; JQ728081; JQ728017
*Cx. vagans*	Site 1, Neimeng	JQ728101
*Cx. modestus*	Site 1, Neimeng	JQ728108; JQ728109; JQ728110; JQ728111; JQ728112
	Site 3, Shanxi	JQ728375; JQ728376
	Site 2, Xinjiang	JQ728296
*Cx. tritaeniorhynchus*	Site 6, Site 8–9, Yunnan	JQ728031; JQ728350; JQ728346; JQ728238
	Site 10–12, Hainan	JQ728059; JQ728060;JQ728061; JQ728062
*Cx. gelidus*	Site 9, Yunnan	JQ728366
*Ae. prominens*	Site 9, Yunnan	JQ728239;JQ728240;JQ728145; JQ728146
*Ae. flavescens*	Site 1, Neimeng	JQ728104; JQ728105; JQ728106; JQ728107
*Ae. dorsalis*	Site 1, Neimeng	JQ728117; JQ728118; JQ728119; JQ728120
	Site 4, Qinghai	JQ728317
	Site 2, Xinjiang	JQ728281; JQ728282; JQ728283
*Ae. omorii*	Site 9, Yunnan	JQ728272
*Ae. fengi*	Site 5, Zhejiang	JQ728015
*Ae. albolateralis*	Site 10, Hainan	JQ728394; JQ728395
	Site 7, Guizhou	JQ728365
	Site 9, Yunnan	JQ728289
*Ae. khazani*	Site 7, Guizhou	JQ728364
*Ae. desmotes*	Site 7, Guizhou	JQ728361
*Ae. tonkinensis*	Site 7, Guizhou	JQ728360
*Ae. japonicus*	Site 6, Yunnan	JQ728181
	Site 5, Zhejiang	JQ728068; JQ728069
*Ae. albolineatus*	Site 10, Hainan	JQ728308
*Ae. chrysolineatus*	Site 9, Yunnan	JQ728271
*Ae. formosensis*	Site 7, Guizhou	JQ728362; JQ728363
	Site 9, Yunnan	JQ728260; JQ728153
*Ae. elsiae*	Site 9, Yunnan	JQ728332
	Site 5, Zhejiang	JQ728093; JQ728094
*Ae. togoi*	Lab	JQ728038; JQ728039
*Ae. vexans*	Site 11, Hainan	JQ728135; JQ728136; JQ728137; JQ728049
	Site 1, Neimeng	JQ728095; JQ728096;JQ728097; JQ728098; JQ728099
	Site 9, Yunnan	JQ728392; JQ728393
	Site 2, Xinjiang	JQ728287; JQ728288
*Ae. kasachstanicus*	Site 2, Xinjiang	JQ728276; JQ728277; JQ728278
*Ae. aegypti*	Site 8, Yunnan	JQ728344; JQ728345
	Lab	JQ728041
*Ae. novoniveus*	Site 7, Guizhou	JQ728368; JQ728369
*Ae. dissimilis*	Site 9, Yunnan	JQ728018; JQ728385; JQ728384; JQ728259; JQ728258
*Ae. craggi*	Site 5, Zhejiang	JQ728142; JQ728143
*Ae. niveoides*	Site 8, Yunnan	JQ728201
*Ae. annandalei*	Site 8–9, Yunnan	JQ728202; JQ728227
*Ae. subsimilis*	Site 8, Yunnan	JQ728226
*Ae. aureostriatus kanaranus*	Site 9, Yunnan	JQ728225
*Ae. gilli*	Site 9, Yunnan	JQ728215; JQ728216
*Ae. albopictus*	Site 10–12, Hainan	JQ728063; JQ728064; JQ728065; JQ728066; JQ728067;JQ728299 JQ728300; JQ728301
	Site 7, Guizhou	JQ728192; JQ728193; JQ728194
	Lab	JQ728019
*Ae. subalbopictus*	Site 7, Guizhou	JQ728198
*Ae. pseudalbopictus*	Site 7, Guizhou	JQ728197
*Ae. albotaeniatus mikiranus*	Site 9, Yunnan	JQ728248; JQ728249; JQ728250; JQ728251; JQ728154
*Ae. assamensis*	Site 9, Yunnan	JQ728190; JQ728191
	Site 7, Guizhou	JQ728355; JQ728356
*Ae. Vittatus*	Site 10, Hainan	JQ728328
*Ae. mediolineatus*	Site 12, Hainan	JQ728297
*Ae. malikuli*	Site 9, Yunnan	JQ728324; JQ728325; JQ728326
*Ae. harveyi*	Site 8–9, Yunnan	JQ728211; JQ728351; JQ728352; JQ728353
*Ar. flavus*	Site 9, Yunnan	JQ728321; JQ728322; JQ728323
*Ar. durhami*	Site 9, Yunnan	JQ728171; JQ728172; JQ728173; JQ728174; JQ728175; JQ728331
*Ar. subalbatus*	Site 6, Yunnan	JQ728219
	Lab	JQ728033
*Hz. proxima*	Site 9, Yunnan	JQ728213; JQ728214
*Hz. menglianensis*	Site 9, Yunnan	JQ728377
*Hz. lii*	Site 9, Yunnan	JQ728252; JQ728253
*Hz. chengi*	Site 9, Yunnan	JQ728255; JQ728257
*Hz. reidi*	Site 8–9, Yunnan	JQ728182; JQ728183; JQ728184; JQ728254; JQ728256; JQ728217
*Ur. nivipleura*	Site 9, Yunnan	JQ728221; JQ728222
*Ur. macfarlanei*	Site 11, Hainan	JQ728128; JQ728129; JQ728130; JQ728131; JQ728132; JQ728133; JQ728134; JQ728016
	Site 2, Xinjiang	JQ728311
*Ur. lutescens*	Site 9, Yunnan	JQ728165; JQ728335; JQ728334
*Ur.bicolor*	Site 9, Yunnan	JQ728223; JQ728224
*Ur. novobscura*	Site 8, Yunnan	JQ728357
*Ur. jinhongensis*	Site 9, Yunnan	JQ728228; JQ728229
*Tx. gravelyi*	Site 8–9, Yunnan	JQ728144; JQ728341; JQ728330; JQ728210
*Tx. edwardsi*	Site 9, Yunnan	JQ728337
*Tx. splendens*	Site 8, Yunnan	JQ728340; JQ728126; JQ728127
*Tx. kempi*	Site 8, Yunnan	JQ728329
*Tx. aurifluus*	Site 9, Yunnan	JQ728204
*Tr. aranoides*	Site 8–9, Yunnan	JQ728166; JQ728167; JQ728168; JQ728169; JQ728170; JQ728262; JQ728263
*Tr. tarsalis*	Site 5, Zhejiang	JQ728014
	Site 7, Guizhou	JQ728371
*Tr. similis*	Site 7, Guizhou	JQ728367; JQ728320
*Ml. jacobsoni*	Site 9, Yunnan	JQ728185; JQ728186; JQ728187; JQ728273
*Ml. genurostris*	Site 10, Hainan	JQ728046
*Cq. crassipes*	Site 9, Yunnan	JQ728179
	Site 10–11, Hainan	JQ728121; JQ728122; JQ728123; JQ728124; JQ728125; JQ728319
*Cq. richiardii*	Site 2, Xinjiang	JQ728309; JQ728310
*Cs. nipponica*	Site 2, Xinjiang	JQ728316
	Site 1, Neimeng	JQ728100
*Cs. annulata*	Site 2, Xinjiang	JQ728312; JQ728313; JQ728314; JQ728315
*Ma. uniformis*	Site 8–9, Yunnan	JQ728176; JQ728177; JQ728178
	Site 10–12, Hainan	JQ728055; JQ728056; JQ728047; JQ728048; JQ728318
*Mi. luzonensis*	Site 9, Yunnan	JQ728156; JQ728157
*Or. anopheloides*	Site 12, Hainan	JQ728138; JQ728139; JQ728140
*To. houghtoni*	Site 9, Yunnan	JQ728195; JQ728196; JQ728275

### Sequence Analysis

Individual species were represented by one to eight individuals giving a total of 404 COI sequences, representing 122 species and subspecies. We identified and excluded 3 pseudogenes from further analyses by only selecting sequences without insertions, deletions and stop codons. COI sequences contain a large number of A+T pairs (average of 69% for all codons), particularly at the third codon position (93.4%) (Table S1). There was, however, no G content in *Orthopodomyia anopheloides* and *Topomyia houghtoni* at the third codon. As in the case of *Drosophila* [20], [21], this quite strong bias is apparently caused by the relative abundance of iso-accepting tRNA. All sequences contained less T in the first codon compared to the second. However, the A content of the first codon was higher than that of the second. The average *R*-value (transitions/transversions) was 0.7.

### Neighbor-Joining (NJ) Tree

**Figure 2:**
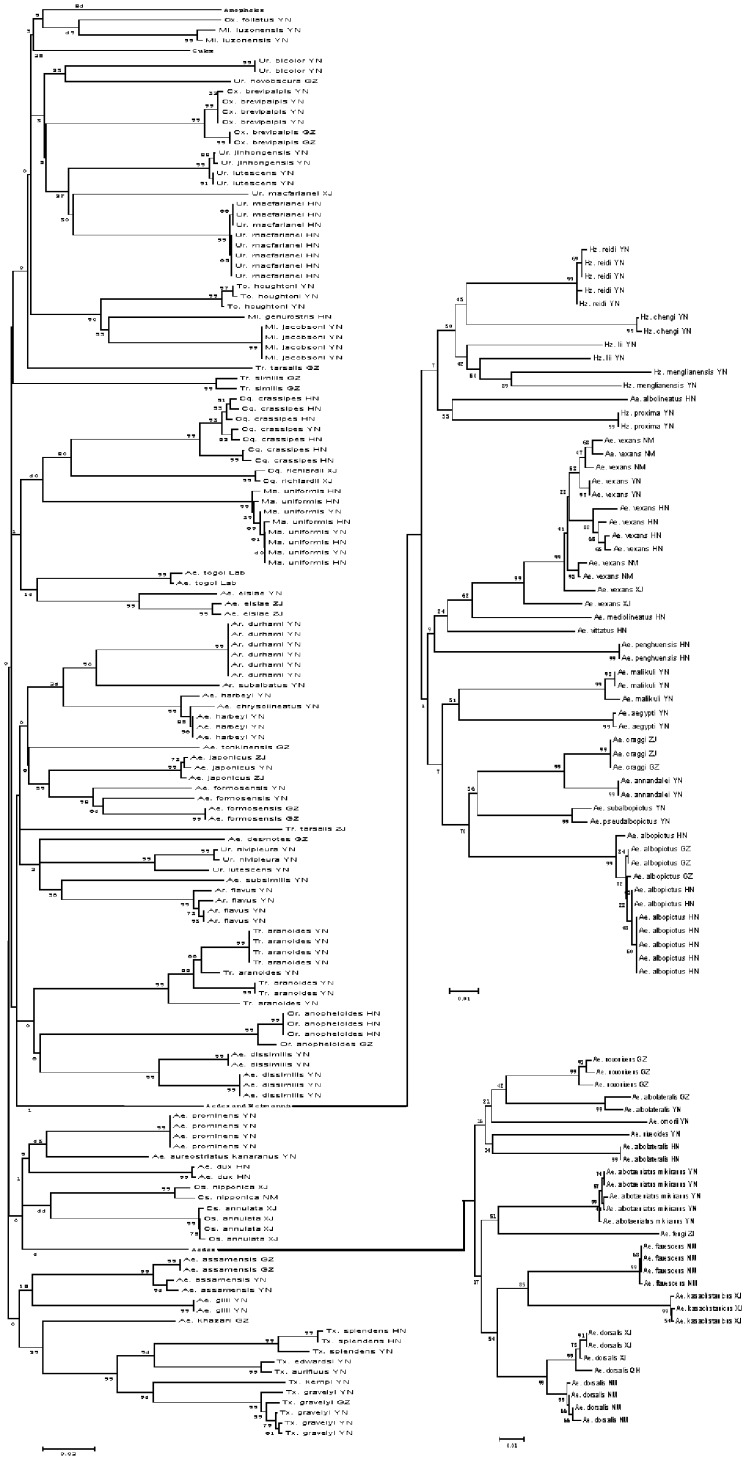
NJ phylogenetic tree based on Kimura two-parameter genetic distances of COI gene sequences of mosquitoes prevalent in China. Sequence analysis was conducted using MEGA version 4.0 software with 1000 replications. Most major branches on the tree represent recognized groups, including all genera and subgenera except *Anopheles* and *Culex* which comprise separate subtrees and are shown in detail in Fig.3.

**Figure 3:**
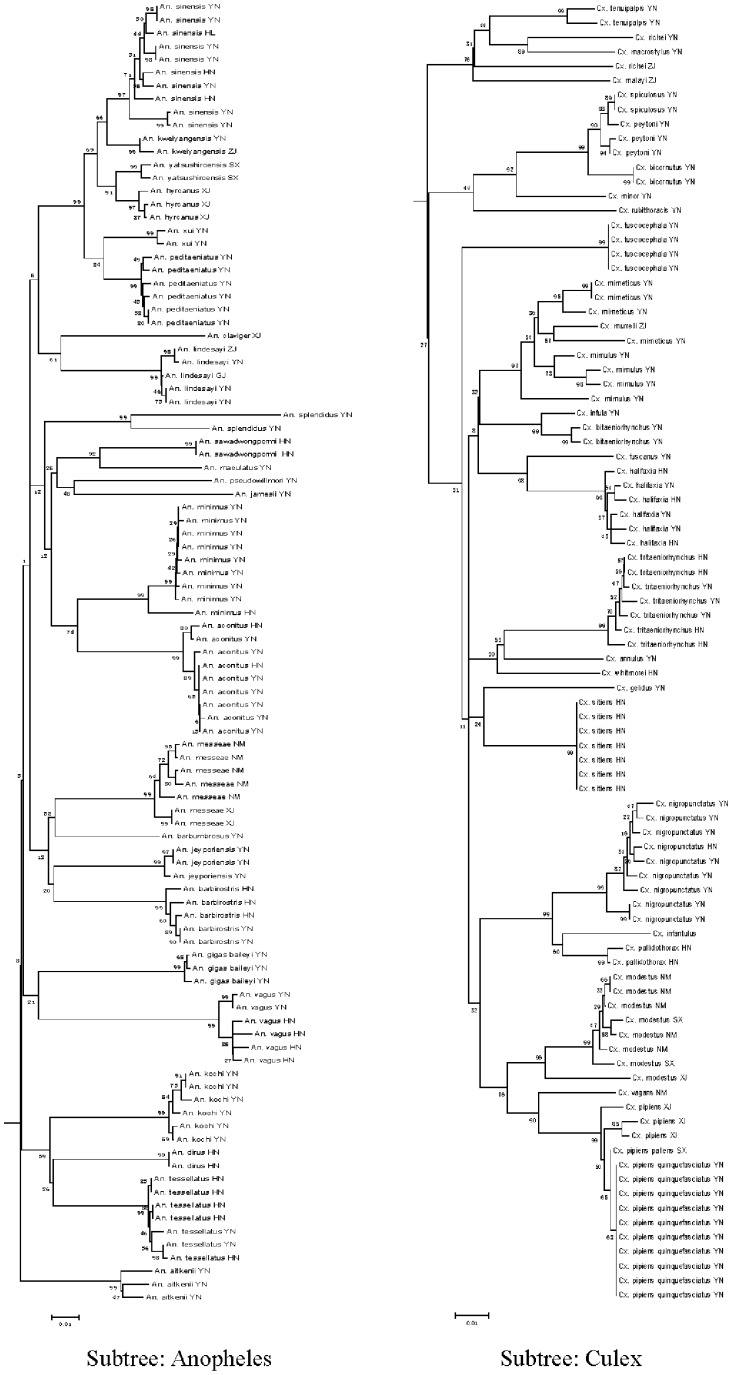
Two distinct sub-trees comprised of *Anopheles* and *Culex* in the NJ phylogenetic tree (Fig. 2).

The Neighbor-Joining (NJ) tree method is conceptually related to clustering, but without the assumption of clock-like behavior [22]. COI gene fragments accurately revealed species boundaries and provided a clear phylogenetic signal (Figs. 2 and 3). Most of the major branches on the tree represent distinct taxonomic groups, including all genera and subgenera. Moreover, specimens of the same species always grouped closely together, regardless of collection site, and, except for some specimens from Hainan Island, no obvious geographic differences in sequences within the same species were found.

Combining NJ tree and bootstrap analysis is the most appropriate method for evaluating phylogenetic trees using distance methods [23]. Nodes linking sequences of individuals of the same species had a high bootstrap value (98%–99%) whereas some linking sequences of geographically different individuals had low bootstrap values (6%–99%).

### Species Boundaries

**Figure 4:**
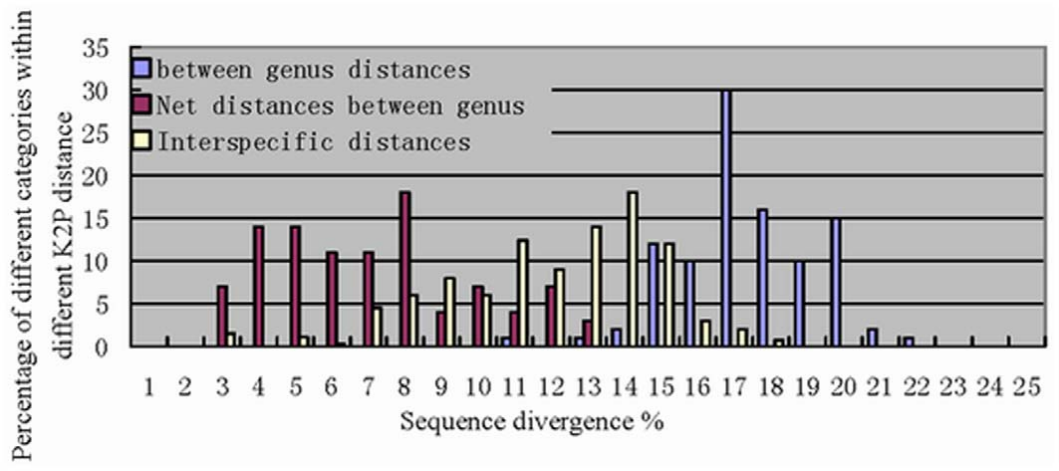
Pairwise comparisons between COI sequences among mosquito species separated into three categories; interspecific distances, between gene distances and net distances between genera. All sequences were grouped with MEGA software, each group includes all species of a particular genus.

All species had a distinct set of COI sequences. Excluding the *Culex mirneticus* subgroup and the species listed in Table 2 (see Discussion section), most (98%) conspecific sequences showed <2% (range  = 0% to 1.67%), whereas >98% of interspecific divergence was in specimens with >2% K2P divergence (range  = 2.3% to 21.8%). Sequence divergence was even higher among species in different genera, ranging from 10.9% to 21.8% (Fig. 4).

**Table 2:** Intraspecific K2P distance, transversion distance, and morphological characteristics of some mosquitoes.

Species	K2P distance (%)	Transversion distance (%)	Variation in morphological characters
*A*. *dorsalis*	2.98	1.11	stripe shape and color of metascutellum
*A*. *vexans*	4.71	1.86	mesopleuron and urotergite
*T*. *aranoides*	5.72	1.29	stable
*T*. *splendens*	2.79	1.29	stable
*C*. *modestus*	4.71	1.67	larvae chest hair and male terminalia
*C*. *crassipes*	3.57	0.37	stable
*A*. *sinensis*	2.61	0.18	stable

**Figure 5:**
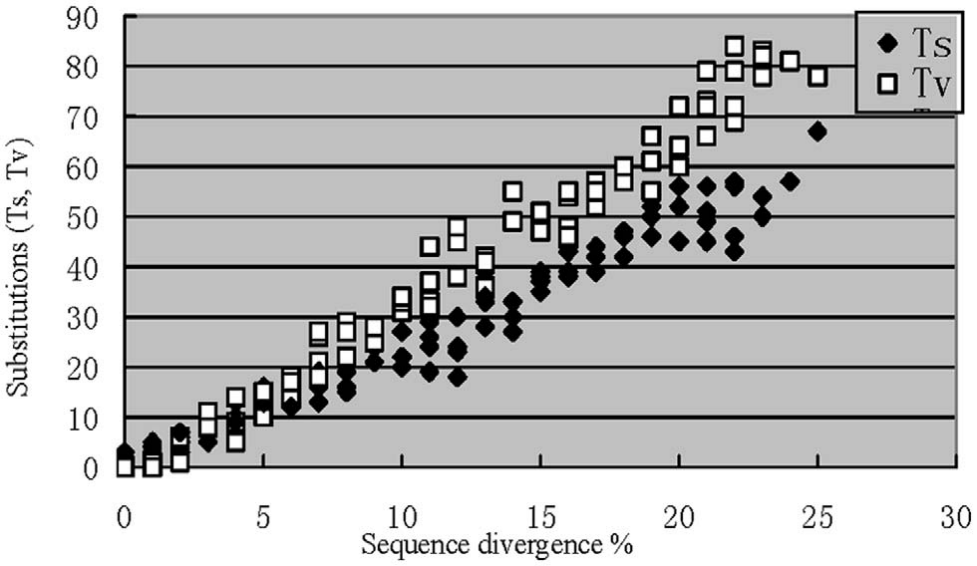
The numbers of COI transitions (ts) and transversions (tv) plotted against sequence divergence.

Transition and transversion distances varied consistently with sequence divergence (Fig. 5). Transition distance was significantly greater than transversion distance when sequence divergence was <2%. However, transversion distances increased slowly with sequence divergence to eventually exceed transition distances at K2P divergence of ≥6%. Both transition and transversion distances then decreased until K2P divergence reached about 15%. The relationship between the transversion distance, sequence divergence, and morphological characteristics are shown in Tables 2 and 3.

**Table 3:** Interspecific K2P distance, transversion distance, and morphological characters of some mosquitoes.

Species	K2P distance (%)	Transversiondistance (%)	Variation in morphological characters
*Ae*. *craggi* and *Ae*. *annandalei*	2.99	0.37	male terminalia
*Cx*. *minor* and *Cx*. *spiculosus*	1.86	0.37	male antenna and terminalia

## Discussion

### Accuracy of COI

The primary function of DNA barcoding is accurate species identification. We found that COI sequence differences among congeneric mosquito species were approximately 30 times higher than the average differences within species. Moreover, more than 98% of COI fragments had clear interspecific boundaries, a result consistent with the results of other authors [13]. The average conspecific K2P divergence in this study, 0.39%, is similar to values reported for fish species in Australia [24] and slightly higher than those reported for North American birds (0.27%) [25] and moths (0.25%) [10]. It is slightly less than the K2P divergence value reported for Canadian mosquitoes (0.55%) [13].

### Transversion Distance and Speciation

Mitochondrial DNA (mtDNA) functions as a molecular clock in that transversions accumulate in a linear fashion over time [26], [27]. Comparison of the molecular and morphological data indicates that the number of transversions may raise to about 7 value without apparent or detectable changes in morphology. (Fig. 5). Transition distance was significantly greater than transversion distance when sequence divergence was below 2% at which level there were almost no morphological differences between specimens. At higher levels of sequence divergence transversion distances slowly increased, eventually exceeding transition distances when sequence divergence reached 6%. Morphological differences were undetectable when sequence divergence was about 2% but were distinct when this reached 6%. Transversion distances increased steadily at sequence divergence levels of 6% to 15% at which level plesiomorphy also first became evident. Plesiomorphy stabilized at sequence divergence of 15%. In addition, the vast majority of intraspecific distances occurred between sequence divergence levels of 6% and 15% whereas most intergeneric distances occurred from 15% to 20% (Fig. 4). Very few intraspecific, and no intergeneric, distances occurred between sequence divergence levels of 2% and 6%.

We found that transversion distances indicated a clear boundary between species. The transversion distance between most species was <1.1% at sequences divergence values of less than 2%. There were, however, some exceptions; although the transversion distance between two plesiomorphous species was usually <1.1% (Table 3), some species with anomalous intraspecific COI sequences divergences >2% (Table 2) had intraspecific transversion distances >1.1%. This suggests the presence of cryptic species, which, if confirmed, in turn suggests that transversion distances may be a useful supplement to barcoding information in species identification**.** Further research on the use of transversion as an additional index of taxonomic similarity is recommended.

### Molecular Data Versus Morphology

Sequence divergence values of 14% to 16% were indicative of either interspecific or intergeneric differences. There are two possible reasons for this; temporary substitution saturation of the COI fragment and the limitations of morphological identification.

We found some cases of high intraspecific sequence divergence among *Aedes dorsalis*, *Aedes vexans*, *Culex modestus*, *Tripteroides aranoides*, and *T*oxorhynchites *splendens* (Table 2). Although the degree of niche separation within these species remains unclear, this result suggests the existence of cryptic species. We also detected intraspecific sequence divergence slightly greater than the 2% threshold within *Coquillettidia crassipes* and *Anopheles sinensis* (Table 2). Although no morphological differences within these species were observed, differences in feeding habits and habitat have been documented within *Anopheles sinensis* populations[19]. This, together with the >2% level of COI sequence divergence, suggests the presence of cryptic species [28]. Some cases of low interspecific sequence divergence were found among some pairs of species (Table 3), including *Aedes craggi* and *Aedes annandalei*, as well as *Culex spiculosus* and *Culex minor*. Although there is no evidence of niche separation between these species, slight morphological differences were observed. This suggests that the taxonomic status of these species should be re-confirmed. Although few doubt that mtDNA barcodes are a valuable molecular tool for matching unidentified specimens to described taxa, there has been relatively little use of barcodes to delimit species [29]. More research on rDNA, morphology, biogeography and ethology are required to improve the applicability of barcoding to species-level taxonomy.

*Culex neomimulus* was previously classified as *C*ulex *mimulus* in the *C*ulex *mirneticus* group [30]. Although our COI data supports the previous view, we found that anomalous COI sequence divergence values were relatively common in the *C*ulex *mirneticus* group with some morphologically distinct specimens having similar barcodes. This could be due to infection with the *Wolbachia* bacteria. The maternally inherited *Wolbachia* bacteria causes a loss of haplotype diversity in populations by inducing a selective sweep of the initially infected individual's haplotype through a population. We detected *Wolbachia* infection in *Culex mimulus* so it’s possible that this may also occur in this species. Although Smith et.al concluded that the presence of *Wolbachia* DNA in total genomic extracts is unlikely to compromise the accuracy of the DNA barcode library, this is a complex problem that requires further investigation [31].

### Pseudogenes

The presence of pseudogenes can affect the accuracy of barcoding identification but, since their incidence was <1%, their influence on our data was presumably small. The distinctive characteristics of the COI gene (no insertions, deletions and stop codons) allowed pseudogenes to be easily identified and excluded from the sequences we obtained. Although the leakage of paternal mtDNA may influence the results of barcoding this phenomenon is only occasionally (<0.004%) found in higher animals.

A total of three pseudogenes were detected. For instance, one of the samples of *A*edes *dissimilis* collected from the same area exhibited high interspecific sequence (3.74%) and transversion divergence (3.00%). A total of 12 different protein sequence sites were observed, which is very rare in the Culicidae. The substitution rate at nucleotide codons 1, 2, and 3 was 1∶2:2, very different to the average of 5∶1:18. We also amplified the pseudogenes of *Uranotaenia lutescens* and *Culex halifaxia*, which have insertions and deletions, respectively. The sequence divergence between pseudogenes and COI fragments in *Culex halifaxia* was 10.93% and the substitution rate at nucleotide codons 1, 2, and 3 was 5∶4:11. The divergence time formula of mtDNA and pseudogenes [32] suggests that the nuclear transfer event occurred 500 million years ago in *Culex halifaxia* and 170 million in *Aedes dissimilis*. We found an insertion site at 54 bp in the sequence of *Uranotaenia lutescens*, with a substitution rate at nucleotide codons 1, 2, and 3 of 7∶1:18. Two different protein sequence sites were also observed. These abnormal phenomena disappeared when the inserted site was deleted manually. Therefore, these anomalous sequences likely caused by the frameshift mutations of PCR.

Overall, DNA-based species identification systems depend on the ability to distinguish intraspecific from interspecific variation. This analysis of 404 COI sequences from 15 mosquito genera and 122 species and subspecies indicates that >98% of specimens formed distinctive clusters and that barcode divergence was relatively large between these groupings. Although it has limitations, DNA barcode technology has several advantages over traditional taxonomic methods as a tool for species identification. For example, it is unaffected by morphological variation between different life cycle stages. Another benefit is that it allows the homogenization, or calibration, of the taxonomic units identified in different areas. DNA barcode technology generally produces accurate results thereby greatly reducing the need for experienced taxonomists.

In summary, this study provides the first COI barcodes for mosquitoes in China and provides further evidence of the effectiveness of DNA barcoding in identifying recognized species. An insufficient number of specimens prevented in-depth investigation of sibling species complexes but we plan to address this area in the future. Care must be taken to exclude pseudogenes from COI databases to ensure the accuracy of molecular identification. COI databases also need to include specimens of the same species collected from different geographical locations in order to determine the extent of intraspecific variation. A complete evaluation of the effectiveness of DNA barcoding for the Culicidae can be achieved through multinational research.

## Materials and Methods

### Ethics Statement

No specific permits were required for this study. All experiments were conducted within state-owned land in China. Therefore, the local ethics committee deemed that approval was unnecessary.

### Mosquito Collections

Mosquito specimens used for constructing DNA barcodes were collected from different Chinese Provinces in 2009 and 2010. Details on specimens collected are provided on Fig. 1 and Table. 1. Larval and adult mosquitoes were collected in the field. Adults were sampled with CO_2_-baited miniature light traps. Larvae were reared individually and associated larval and pupal skins were mounted. All specimens were identified using standard taxonomic keys [19].

### Target Gene Preparation

Total DNA (100 µL to 150 µL) was extracted from each specimen using the Universal Genomic DNA Extration Kit (Invitrogen). PCR was performed to amplify the 5′ COI region of mtDNA using the following cycle: An initial denaturation of 1 min (94°C) followed by five cycles of 94°C for 40 s (denaturation), 45°C for 40 s (annealing), and 72°C for 1 min (extension); 30 cycles of 94°C for 40 s (denaturation), 51°C for 40 s (annealing), 72°C for 1 min (extension) and a final extension at 72°C for 5 min. PCR cocktails were made as follows: A 50 µL solution comprised of 0.3 µL Taq DNA polymerase (5 U/µL), 5 µL of 10×PCR buffer, 5 µL of 2 mmol/L dNTP, 2 µL of 10 µmol/L each of the forward and reverse primers, 5 µL of template DNA and sufficient ddH_2_O to make up to 50 µL. The primer pairs LCO1490 and HCO2198 [33] were used to amplify a 650 bp fragment of COI. The amplified fragments were run on a 1% agarose gel to check the integrity of the fragments after which the PCR product was purified with a normal PCR purification kit (Tiangen). Both reads (forward as well as reverse primer) were done.

### Data Analysis

DNA sequences were aligned using Clustal X [34]. Sequence analysis and Ts/Tv calculation was conducted using MEGA version 4.0 software [14]. Sequence divergence and Ts, Tv distance among individuals was quantified using the Kimura two-parameter distance model [35]. An NJ tree of K2P distances was created to provide a graphic representation of the clustering pattern among different species [36].

## Supporting Information

Table S1 **Sequence divergence and nucleotide composition for the mosquito genera.** The frequencies of nucleotides in sequence are presented as the total average values for all Condon positions and for each condon position separately with the accuracy to tenths of a percent. (*) Figures in brackets are the number of mosquito species used to estimates of sequence divergence for the genus
(PDF)
