# Supplementary material for: Identifying the Main Mosquito Species in China Based on DNA Barcoding
Source: PLoS One. 2012 Oct 10;7(10):e47051. doi: 10.1371/journal.pone.0047051 (PMC3468562; doi:10.1371/journal.pone.0047051)
Supplement: Table S1 — Sequence divergence and nucleotide composition for the mosquito genera. The frequencies of nucleotides in sequence are presented as the total average values for all Condon positions and for each condon position separately with the accuracy to tenths of a percent. (*) Figures in brackets are the number of mosquito species used to estimates of sequence divergence for the genus (PDF) [file pone.0047051.s001.pdf]

Table S1. Sequence divergence and nucleotide composition for the mosquito genera. The frequencies of nucleotides in sequence are presented as the total average values for all Condon positions and for each condon position separately with the accuracy to tenths of a percent. (*) Figures in brackets are the number of mosquito species used to estimates of sequence divergence for the genus.

		Total					First codon position					Second codon position					Third codon position				
Sequence diergence		%	%	%	%	%	%	%	%	%	%	%	%	%	%	%	%	%	%	%	%
Genus (*)	%	T	C	A	G	A+T	T	C	A	G	A+T	T	C	A	G	A+T	T	C	A	G	A+T
Aedes (32)	2.3~17.7	38.6	16.5	29.8	15.1	68.4	27.0	16.7	29.3	27.0	56.3	43.7	26.1	14.1	16.1	57.8	45.0	6.7	46.1	2.2	91.1
Anopheles (26)	2.5~13.6	38.9	15.4	30.0	15.7	68.9	27.6	15.3	27.7	29.5	55.3	43.1	26.5	14.2	16.2	57.3	46.0	4.5	48.0	1.5	94.0
Culex (30)	1.8~15.0	40.0	15.6	29.4	14.9	69.4	27.3	16.9	28.4	27.4	55.7	44.1	25.7	14.1	16.0	58.2	48.6	4.3	45.7	1.4	94.3
Armigeres (3)	2.9~13.0	40.7	16.3	28.9	14.1	69.6	28.4	16.3	29.7	25.6	58.1	43.5	27.3	14.0	15.2	57.5	50.4	5.2	42.9	1.6	93.3
Toxorhynchites (5)	11.1~14.3	40.5	16.3	29.8	13.4	70.3	26.1	18.0	31.7	24.2	57.8	44.6	24.6	15.5	15.3	60.1	50.7	6.1	42.4	0.8	93.1
Uranotaenia (2)	13.2	40.6	14.9	29.5	15.1	70.1	28.2	15.4	28.7	27.7	56.9	44.4	25.2	14.2	16.2	58.6	49.3	3.9	45.6	1.2	94.9
Tripteroides (2)	14.4	38.9	18.0	27.1	15.9	66.0	25.8	17.4	29.2	27.5	55.0	43.5	26.6	14.0	15.9	57.5	47.3	9.9	38.4	4.3	85.7
Coquillettidia		38.5	16.6	29.5	15.5	68.0	27.5	16.9	28.0	27.5	55.5	43.5	25.6	14.5	16.4	58.0	44.4	7.2	45.9	2.4	90.3
Culiseta		39.6	15.3	29.6	15.4	69.2	26.3	17.4	29.6	26.8	55.9	43.5	25.2	14.0	17.3	57.5	49.1	3.3	45.3	2.3	94.4
Heizmannia		39.3	17.9	27.4	15.5	66.7	25.1	17.4	29.5	28.0	54.6	43.0	27.5	13.0	16.4	56.0	49.6	8.7	39.6	1.9	89.2
Malaya		41.4	15.8	28.0	14.8	69.4	28.5	15.5	29.5	26.6	58.0	43.0	26.1	15.0	15.9	58.0	52.7	5.8	39.6	1.9	92.3
Mansonia		39.6	16.1	29.8	14.5	69.4	27.5	15.9	29.5	27.1	57.0	44.4	25.6	14.5	15.5	58.9	46.9	6.8	45.4	1.0	92.3
Mimomyia		39.5	15.9	30.1	14.5	69.6	26.6	15.9	30.9	26.6	57.5	43.0	26.6	14.0	16.4	57.0	48.8	5.3	45.4	0.5	94.2
Orthopodomyia		36.7	17.4	31.7	14.1	68.4	23.9	19.4	30.3	26.4	54.2	43.0	26.0	15.0	16.0	58.0	43.3	7.0	49.8	0	93.1
Topomyia		42.4	14.8	28.2	14.5	70.6	31.8	12.6	28.5	27.1	60.3	43.9	25.7	14.0	16.4	57.9	51.6	6.1	42.3	0	93.9
